# RACIAL DIFFERENCES IN THE ASSOCIATION BETWEEN LONELINESS AND COGNITIVE STATUS AMONG BLACK AND WHITE MEN

**DOI:** 10.1093/geroni/igac059.1902

**Published:** 2022-12-20

**Authors:** Marino Bruce, Bettina Beech, Gillian Marshall, Roland J Thorpe, Jr.

**Affiliations:** University of Houston Tilman J. Fertitta Family College of Medicine, Houston, Texas, United States; University of Houston, Houston, Texas, United States; University of Washington, Seattle, Washington, United States; Johns Hopkins University, Baltimore, Maryland, United States

## Abstract

**Background:**

Loneliness is a stressor that has been found to increase the likelihood of poor health and dementia. Few studies have focused on this association among men and even fewer studies have examined racial disparities in loneliness and cognitive functioning among this group. The purpose of this study was to examine racial differences in the association between loneliness and cognitive functioning among national sample of men aged 50 years and older.

**Methods:**

Data were drawn from Black and White men in the 2016 Health and Retirement Study who completed the Leave Behind Questionnaire (n=2226). Cognitive function was the primary outcome and was measured by a dichotomous variable derived from a modified version of the Telephone Interview for Cognitive Status. Loneliness was the primary independent variable and was derived from the 3-item UCLA Loneliness Scale.

**Results:**

Black men made up 18.5% of the study sample; however, the proportion of this group with scores indicating cognitive impairment or dementia (35.9%) doubled the corresponding percent of white men (17.6%). Findings from race-stratified modified Poisson regression models indicated that loneliness was associated with a higher prevalence of cognitive impairment or dementia for White men (PR=1.24, CI:1.05-1.47), but Black men (PR=0.92, CI:0.73-1.16).

**Conclusions:**

Results from this study raise important questions about the salience of pooled analyses and suggest a need for tailored approaches to mitigate cognitive decline. Additional studies focusing on Black men are needed to develop effective interventions preserving cognitive functioning among this population.

